# Usefulness of the d-dimer to albumin ratio for risk assessment in patients with acute variceal bleeding at the emergency department: retrospective observational study

**DOI:** 10.1186/s12873-022-00696-4

**Published:** 2022-07-25

**Authors:** Jun Seok Seo, Yongwon Kim, Yoonsuk Lee, Ho Young Chung, Tae Youn Kim

**Affiliations:** 1 Department of Emergency Medicine, Dongguk University Ilsan Hospital, Dongguk University College of Medicine, 27, Dongguk-ro, Ilsandong-gu, Goyang-si, Gyeonggi-do Republic of Korea; 2 Department of Medical Informatics, Kyungpook National University School of Medicine, Daegu, Republic of Korea; 3 Department of Emergency Medicine, Yonsei University Wonju College of Medicine, Wonju, Republic of Korea

**Keywords:** Esophageal and Gastric Varices, Risk assessment, D-dimer, albumin

## Abstract

**Background:**

Acute variceal bleeding (AVB) is a severe complication of portal hypertension that is caused by rupture of the esophageal or gastric varix. Scoring system for risk stratification of AVB is difficult to use because various variables must be entered, and it is difficult to apply early in the emergency department (ED). We compared and analyzed the usefulness of the D-dimer to albumin ratio (DAR) for risk stratification of AVB.

**Methods:**

In this retrospective observational study, medical records of patients with AVB Between January 2019 and December 2020 were assessed. The primary endpoint was to evaluate whether DAR was a predictor of clinical outcomes for AVB. Receiver operating characteristic (ROC) curves were constructed using cut-off values determined by the Youden Index. Univariate and multivariate logistic regression analyses were performed to assess the factors contributing to the development of outcomes.

**Results:**

Overall, 67 patients required intensive care. The cut-off value of DAR for patients requiring intensive care was 400. A DAR > 400 (adjusted HR: 5.636 [95% CI: 2.216–14.332]) independently predicted the need for ICU admission in these patients. Overall, 13 patients required long-term hospitalization. The cut-off value of DAR for patients requiring long-term hospitalization was 403. A DAR > 403 (adjusted HR: 9.899 [95% CI: 2.012–48.694]) independently predicted the need for long-term hospitalization. Overall, 95 patients required transfusion. The cut-off value of DAR for patients requiring transfusion was 121. A DAR > 121 (adjusted HR: 4.680 [95% CI: 1.703–12.862]) independently predicted the need for transfusion. Overall, 11 patients died during study period. The cut-off value of DAR for mortality was 450. A DAR > 450 (adjusted HR: 26.261 [95% CI: 3.054–225.827]) independently predicted mortality.

**Conclusions:**

The DAR can be used for outcome assessment in patients with AVB with various scoring systems, but its explanatory power is not high.

**Supplementary Information:**

The online version contains supplementary material available at 10.1186/s12873-022-00696-4.

## Introduction

Acute variceal bleeding (AVB) is a severe complication of portal hypertension that is caused by rupture of the esophageal or gastric varix [1]. The prognosis of patients with AVB is associated with liver-disease severity [2]. The Child–Pugh and model end-stage liver disease (MELD) scores have been traditionally used [3]. The Child–Pugh score predicts life expectancy and assesses the mortality percentage in patients with cirrhosis [4]. The MELD score is also used together with the Child–Pugh score to assess the percentage of mortality [5]. In addition, the AIMS65, Glasgow-Blatchford (GBS), and Rockall scores are usually used in patients with non-variceal bleeding, which can also be applied to AVB and for risk stratification [6]. The AIMS65 score includes albumin level, international normalized ratio (INR), mental status, systolic blood pressure, and age > 65 years and is used to predict the percentage of mortality, length of stay, and cost of admission from upper gastrointestinal bleeding [7]. The Rockall score is divided into pre-endoscopy and post-endoscopy. The pre-endoscopy Rockall score is used to predict mortality before endoscopy [8]. The Glasgow-Blatchford score is graded 0 for outpatients, and is used to evaluate the risk of transfusion and endoscopy or surgery if it is higher than 0 [9]. However, this scoring system is difficult to use because various variables must be entered, and it is difficult to apply early in the emergency department (ED) [10]. In addition, as the outcome is different for each scoring system, it is difficult for clinicians to use different scores to determine whether or not to admit the patient to the intensive care unit (ICU) and to predict transfusion or the hospitalization period and long-term mortality [11]. An increased D-dimer value has been reported to be independently associated with the risk of death from various diseases [12]. In particular, it was reported that coagulation activation and hyperfibrinolysis were more activated in patients with variceal bleeding than in patients without, and it was found that higher a d-dimer value was a significant predictor of death in patients with variceal bleeding [13]. In addition, serum albumin levels were also reported to be associated with prognosis in patients with sepsis, non-variceal upper gastrointestinal bleeding, and liver cirrhosis [14]. Low albumin levels in non-variceal upper gastrointestinal bleeding were found to reflect the severity of bleeding and rate of complications [15]. Hypoalbuminemia was found to be associated with hypercoagulation [16, 17]. In addition to hypercoagulation, which is the cause of high d-dimer levels, low albumin is a predictive index of severity in patients with coronavirus disease. A relatively new marker, the d-dimer to albumin ratio (DAR), was found to be a predictor of mortality [18].

The purpose of this study was therefore to evaluate the usefulness of the DAR, which can be calculated more easily than other validated scoring systems, in predicting the need of patients with AVB for intensive care, long-term hospitalization, and transfusion and in assessing their mortality. To that end, we analyzed outcomes based on patient records and compared our results with those based on other scoring system.

## Materials and methods

This was a retrospective observational study performed in the ED of a tertiary university hospital visited by 43,000 patients annually, with approximately 60 patients presenting with AVB. This study was approved by the Institutional Review Board of Wonju Severance Christian Hospital (IRB No. CR321093). The study protocol conformed to the ethical guidelines of the Declaration of Helsinki (1975) and its later amendments. As the study involved retrospective and observational analysis, the requirement for informed consent was waived, and patient records and information were anonymized before analysis. Computerized hospital records were reviewed, and any patients for whom “esophageal varices with hemorrhage (I85.01)” based on the International Classification of Diseases, 10th revision coding, was used as a discharge code were initially considered for study selection. Included were patients diagnosed with “variceal bleeding” via esophagogastroduodenoscopy after being admitted to the hospital.

### Participants

A total of 522 patients with upper gastrointestinal bleeding visited the emergency department between January 2019 and December 2020, and 137 patients were retrospectively diagnosed with variceal bleeding according to their medical records. Patients who did not undergo laboratory tests were excluded from the analysis.

### Study variables

Data were collected from a retrospective review of patient electronic medical records performed by emergency physicians blind to study objectives and hypotheses. Data on age, sex, hospitalization period, serum albumin level (reference range: 3.5–5.5 g/dL), d-dimer level (reference range: < 250 ng/mL), and variables required for each scoring system were recorded.

The Modified early warning score (MEWS) is scored based on systolic blood pressure, heart rate, respiratory rate, body temperature, and alertness [19]. The Child–Pugh is scored based on bilirubin and albumin levels, the international normalized ratio (INR), the presence or absence of ascites, and the presence or absence of encephalopathy. The MELD score considers dialysis, serum creatinine, bilirubin, INR, and serum sodium level data [20]. The AIMS65 score is based on the albumin, INR, mental status, presence of shock, and age > 65 years. The GBS score is based on hemoglobin and blood nitrogen urea levels, systolic blood pressure, sex, heart rate, clinical presentations, hepatic disease, and cardiac failure. In addition, the Pre-endoscopy Rockall score is based on age, presence of shock, and comorbidities [21]

The collected blood samples were sent to the laboratory for analysis (ADVIA 2120i automated hematology analyzer, Siemens Healthcare Diagnostics Manufacturing Limited, Dublin, Ireland), and the coagulation profile (CS-5100 hemostasis system, Sysmex Corp., Kobe, Japan), D-dimer, and albumin were detected with serum biochemical tests (Dimension VISTA 1500, Siemens, Delaware, USA).

### Study endpoint

The primary endpoint of this study was the efficacy of the DAR in predicting the need for intensive care, long-term hospitalization, transfusion in the ED, and mortality in patients with AVB who visited the ED. The secondary endpoint was the comparative efficacy of the MEWS, MELD, Child–Pugh, GBS, pre-endoscopy Rockall, and AIMS65 scores in predicting the need for intensive care, long-term hospitalization, transfusion, and mortality.

The admission criteria for the ICU are based on priority models [22]. Admission is decided according to clinician judgment, vital signs as objective parameters, and clinical examination and acute onset physical signs when necessary. We categorized patients using the priority model. Priority 1 is considered for patients requiring intensive care and monitoring, such as those on a ventilator or intravenous cardiovascular medications, respiratory failure requiring ventilation after surgery, and those requiring invasive monitoring. Priority 2 is considered for patients who may need immediate treatment at any time during intensive monitoring and chronic disease that can rapidly worsen. Priority 3 is considered for patients with underlying or acute disease, which may require intensive care. Priority 4 is not appropriate for ICU. Long-term hospitalization was defined as hospitalization lasting longer than 14 days [23, 24].

### Statistical analysis

Continuous data are presented as means with standard deviations or medians (interquartile ranges). The normality of data distribution was assessed using the Shapiro–Wilk test. Categorical variables are presented as counts and percentages. Continuous data were analyzed using Student’s t-test or the Mann–Whitney U test, as appropriate. Categorical data were analyzed using a chi-square test or Fisher’s exact test, as appropriate. To assess the predictability of the DAR and MEWS, Child–Pugh, MELD, pre-endoscopy Rockall GBS, and AIMS65 scores, receiver operating characteristic (ROC) curves were constructed using cut-off values determined with the Youden Index [25]. Univariate and multivariate logistic regression analyses were performed to assess the factors contributing to ICU admission. Variables with a *p*-value of < 0.2 in the univariable logistic regression analysis were entered into the multivariable logistic regression analysis. Statistical significance was set at *p* < 0.05 [26]. A Kaplan–Meier type plot was constructed to show estimated survival probabilities from after acute variceal bleeding at 30 days. All analyses were performed using SPSS ver. 23 (IBM Corp., New York, NY, USA) and MedCalc Statistical software version 17.5.3 (MedCalc Software, Ostend, Belgium).

## Results

### General characteristics

**Fig. 1:**
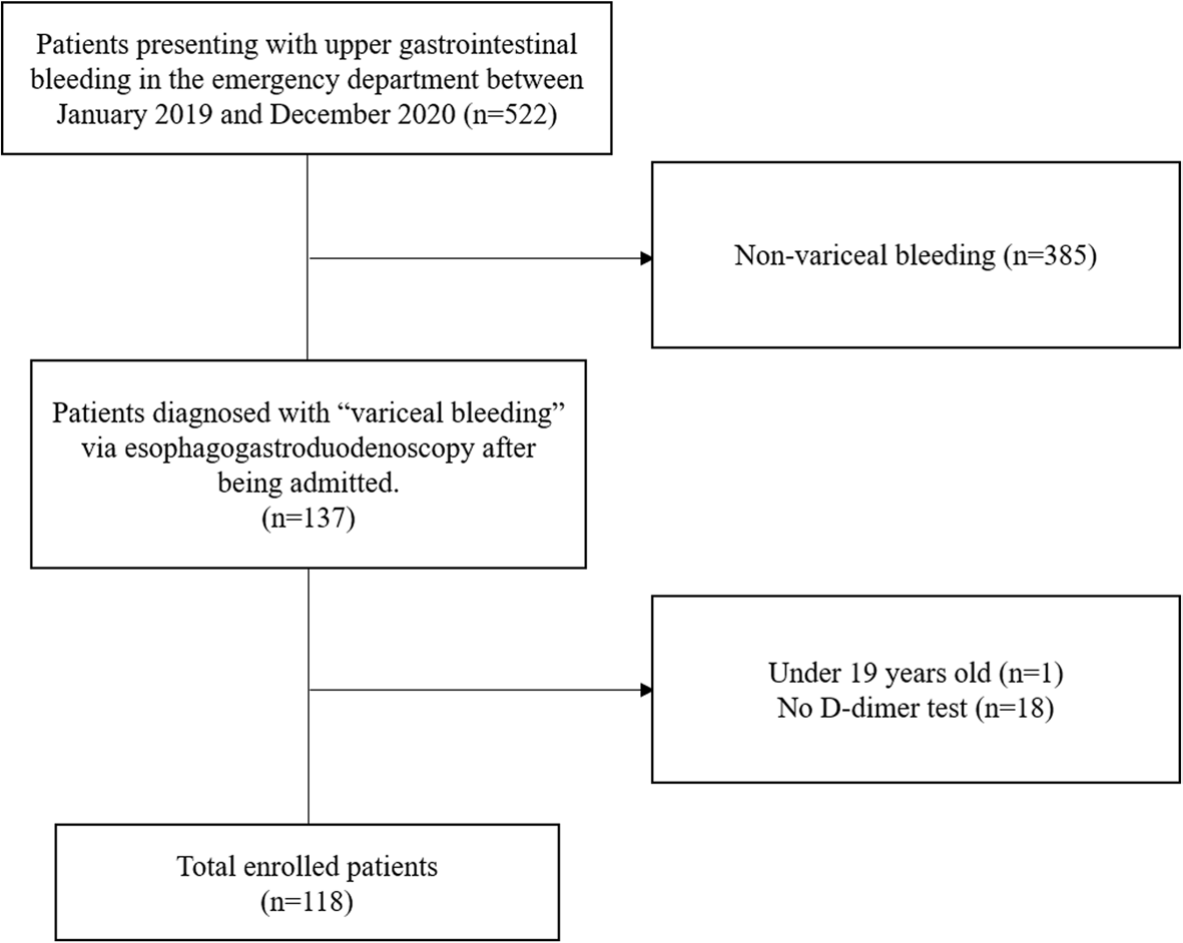
Enrollment chart

Between January 2019 and December 2020, 136 patients with AVB visited the ED. Among them, we excluded patients who had incomplete data (*n* = 18). Thus, 118 patients were included in this study (Fig. 1).

Results related to age, sex, hospitalization period, D-dimer, albumin, MEWS, pre-endoscopy Rockall, AIMS65, GBS, MELD, and Child–Pugh scores are shown in Table 1. The median D-dimer level was 850 (50–17,600) ng/mL, and the median albumin level was 3.1 (1.1–5.7) g/dL. Eighty-nine patients were diagnosed with recent hemorrhage after endoscopy, 67 patients required intensive care, 95 patients required transfusion, 13 patients required long-term hospitalization, and 11 patients died during the study period (Table 1).

**Table 1:** Patient characteristics

	**All patient (*N* = 118)**	**ICU (*N* = 67)**	***P* value**	**Long stay (*N* = 13)**	***P* value**	**Transfusion (*N* = 95)**	***P* value**	**Mortality (*N* = 11)**	***P* value**
Age	60.63 ± 12.66	60.07 ± 13.13	0.455	63.15 ± 14.61	0.527	59.88 ± 13.03	0.151	60.91 ± 12.05	0.810
Male sex, n (%)	96 (81.4%)	57 (85.1%)	0.342	10 (76.9%)	0.954	77 (81.1%)	1.000	10 (90.9%)	0.654
Hospitalization period, day	6 (1–50)	5 (1–49)	0.083	25 (16–49)	0.000	5 (1–49)	0.007	12 (1–25)	0.268
Recent hemorrhage, n (%)	89 (75.4%)	56 (83.6%)	0.032	12 (92.3%)	0.247	72 (75.8%)	1.000	11 (100%)	0.105
MEWS	2.5 (1–10)	3 (1–10)	0.002	4 (1–10)	0.008	3 (1–10)	0.001	4 (1–8)	0.024
MELD score	14 (7–37)	16 (7–37)	0.008	20 (15–37)	0.000	15 (7–37)	0.001	21 (10–37)	0.002
Child–Pugh score	7 (5–14)	8 (5–14)	0.031	11 (7–14)	0.000	7 (5–14)	0.004	11 (6–12)	0.000
Rockall score (Pre-endoscopy)	4 (0–7)	13 (5–18)	0.019	14 (9–17)	0.007	13 (6–18)	0.004	14 (10–16)	0.001
AIMS65 score	1 (0–5)	2 (0–5)	0.000	3 (1–5)	0.000	2 (0–5)	0.000	2 (0–5)	0.002
GBS score	10 (0–15)	4 (2–7)	0.002	5 (3–6)	0.105	4 (2–7)	0.000	5 (4–6)	0.005
Shock Index	0.88 (0.26–1.88)	1.00 (0.26–1.88)	0.001	0.92 (0.35–1.88)	0.310	0.97 (0.26–1.88)	0.000	1.05 (0.26–1.80)	0.071
D-dimer, ng/mL	850 (50–17,600)	1,250 (50–8,500)	0.001	2,100 (250–3,700)	0.008	950 (50–8,500)	0.048	2,050 (550–17,600)	0.011
Albumin, g/dL	3.1 (1.1–5.7)	2.9 (1.1–4.2)	0.004	2.1 (1.6–3.7)	0.001	2.9 (1.1–4.2)	0.000	2.3 (1.1–3.5)	0.002
D-dimer to albumin ratio	282.86 (17.86–6,518.52)	475 (17.86–3,236.84)	0.000	1,093.75 (86.21–1,888.89)	0.001	343.75 (17.86–3,236.84)	0.008	1,093.75 (157.14–6,518.52)	0.001

*ICU* Intensive care unit, *MEWS* Modified early warning score, *MELD* Model for end-stage liver disease, *GBS* Glasgow-blatchford score

### Comparison of the diagnostic accuracy of variables for predicting outcomes

**Fig. 2:**
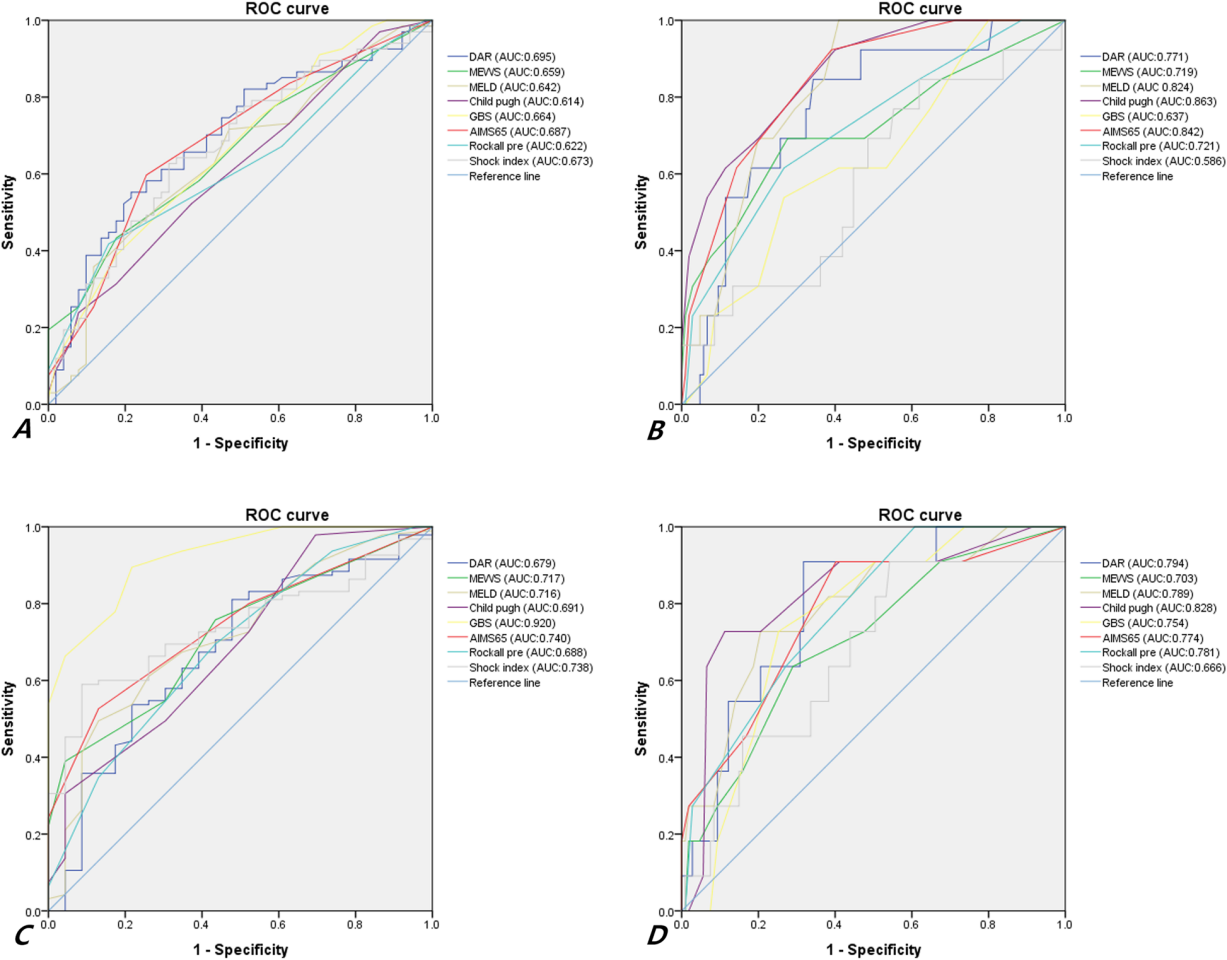
Receiver operating characteristic (ROC) curve for the outcomes. **A** ROC curve analyses for predicting the need for intensive care. **B** ROC curve analyses for predicting the need for long-term hospitalization. **C** ROC curve analyses for predicting the need for transfusion. **D** ROC curve analyses for predicting mortality

The area under the curve of the DAR for need for intensive care was 0.695, with a 95% confidence interval (CI) of 0.604–0.777, which was higher than those of the other scores (Fig. 2A). The area under the curve of the DAR for need for long-term hospitalization was 0.771, with a 95% CI of 0.685–0.843, but the area under the curve of the Child–Pugh score for the same was 0.863, with a 95% CI of 0.787–0.919 (Fig. 2B). The area under the curve of the DAR for need for transfusion was 0.679, with a 95% CI of 0.587–0.762, but the area under the curve of the GBS for the same was 0.920, with a 95% CI of 0.855–0.962 (Fig. 2C). The area under the curve of the DAR for predicting mortality was 0.794, with a 95% CI of 0.709–0.863, but the area under the curve of the Child–Pugh score for the same was 0.828, with a 95% CI of 0.747–0.891 (Fig. 2D). A table regarding the predicting accuracy of the DAR for the outcomes was presented separately as Supplement 1. Supplement 2 depicts the prediction accuracy of the analyzed laboratory factors (D-dimer, albumin, hemoglobin, blood urea nitrogen, creatinine, bilirubin, sodium, and INR) for the different outcomes. There was no single laboratory test with a higher area under the ROC curve in predicting mortality than the DAR. Single D-dimer and albumin levels had a lower area under the ROC curve for need for intensive care than the DAR.

### Univariate and multivariate analysis for predicting outcome assessment

Univariate analysis indicated that a DAR > 400 (crude OR: 4.008 [95% CI: 1.789–8.979]; *p* = 0.001) might be a risk factor for the need for intensive care in AVB. According to the results of the univariate analyses of prognostic factors affecting the need for intensive care in AVB, age, sex, and recent hemorrhage after endoscopic evaluation were incorporated in the multivariate regression analysis. A DAR > 400 (adjusted OR: 5.636 [95% CI: 2.216–14.332]) independently predicted the need for intensive care in patients with AVB. The MELD score was not statistically significant in the multivariate analysis for predicting the need for intensive care. The MEWS, Child–Pugh, GBS, AIMS65, and Pre-endoscopy Rockall scores were statistically significant in the multivariate logistic analysis for predicting the need for intensive care (Table 2). A DAR > 403 (adjusted OR: 9.899 [95% CI: 2.012–48.694]) independently predicted the need for long-term hospitalization. However, the GBS was not statistically significant in the multivariate analysis for predicting the need for long-term hospitalization. The MEWS, MELD, Child–Pugh, AIMS65, and Pre-endoscopy Rockall scores were statistically significant in the multivariate logistic analysis for predicting the need for long-term hospitalization (Table 3). A DAR > 121 (adjusted OR: 4.680 [95% CI: 1.703–12.862]) independently predicted the need for transfusion. The other scores were also statistically significant in the multivariate logistic analysis for predicting the need for transfusion (Table 4). A DAR > 450 (adjusted OR: 26.261 [95% CI: 3.054–225.827]) independently predicted mortality. The other scores were also statistically significant in the multivariate logistic analysis for predicting mortality (Table 5).

**Table 2:** The association of factors for intensive care in patients with variceal bleeding

Factor	Non-ICU (*N* = 51)	ICU (*N* = 67)		Need for intensive care	
				Crude OR (95% CI)	Adjust OR^a^ (95% CI)
D-dimer to albumin ratio ≥ 400, n (%)	12 (23.5%)		37 (55.2%)	4.008 (1.789–8.979)	5.636 (2.216–14.332)
MEWS	2 (1–5)		3 (1–10)	1.470 (1.157–0.867)	1.516 (1.163–1.976)
MELD	12 (8–30)		16 (7–37)	1.086 (1.012–1.166)	1.072 (0.998–1.152)
Child–Pugh score	7 (5–12)		8 (5–14)	1.290(1.044–1.593)	1.26 0(1.017–1.560)
GBS	11 (0–17)		13 (5–18)	1.215 (1.081–1.365)	1.207 (1.071–1.361)
AIMS65 score	1 (0–3)		2 (0–5)	1.896 (1.313–2.738)	2.075 (1.384–3.111)
Rockall Score (Pre-endoscopy)	4 (0–5)		4 (2–7)	1.568 (1.099–2.236)	1.715 (1.160–2.534)
Shock index	0.77 (0.36–1.65)		1.00 (0.26–1.88)	6.723 (1.901–23.779)	7.739 (1.991–30.078)

*ICU* Intensive care unit, *OR* Odds ratio, *CI* Confidence interval, *MEWS* Modified early warning score, *MELD* Model for end-stage liver disease, *GBS* Glasgow-blatchford score

^a^Controlling for centered age, sex, and recent hemorrhage after endoscopy

**Table 3:** The association of factors for long-term hospitalization in patients with variceal bleeding

Factor	Short stay (*N* = 105)	Long stay (*N* = 13)	Need for long-term hospitalization	
			Crude OR (95% CI)	Adjust OR^a^ (95% CI)
D-dimer to albumin ratio > 403, n (%)	37 (35.2%)	11 (84.6%)	10.108 (2.126–48.051)	9.899 (2.012–48.694)
MEWS	2 (1–8)	4 (1–10)	1.588 (1.200–2.102)	1.672 (1.231–2.271)
MELD	13 (7–30)	20 (15–37)	1.192 (1.081–1.313)	1.195 (1.080–1.321)
Child–Pugh score	7 (5–12)	11 (7–14)	2.100 (1.486–2.969)	2.123 (1.474–3.058)
GBS	12 (0–18)	14 (9–17)	1.185 (0.973–1.442)	1.168 (0.959–1.423)
AIMS65 score	1 (0–5)	3 (1–5)	3.171 (1.720–5.846)	3.096 (1.623–5.906)
Rockall Score (Pre-endoscopy)	4 (0–7)	5 (3–6)	2.288 (1.234–4.240)	2.143 (1.120–4.101)
Shock index	0.88 (0.26–1.77)	0.92 (0.35–1.88)	2.686 (0.534–13.495)	4.049 (0.662–24.775)

*ICU* Intensive care unit, *OR* Odds ratio, *CI* Confidence interval, *MEWS* Modified early warning score, *MELD* Model for end-stage liver disease, *GBS* Glasgow-blatchford score

^a^Controlling for centered age, sex, and recent hemorrhage after endoscopy

**Table 4:** The association of factors for transfusion in patients with variceal bleeding

Factor	Non- Transfusion (N = 23)	Transfusion (N = 95)		Need for transfusion	
				Crude OR (95% CI)	Adjust OR^a^ (95% CI)
D-dimer to albumin ratio > 121, n (%)	12 (52.2%)		78 (82.1%)	4.206 (1.591–11.116)	4.680 (1.703–12.862)
MEWS	1 (1–4)		3 (1–10)	1.839 (1.226–2.760)	1.883 (1.228–2.888)
MELD	12 (8–29)		15 (7–37)	1.170 (1.040–1.315)	1.176 (1.042–1.326)
Child–Pugh score	7 (5–11)		7 (5–14)	1.631 (1.146–2.319)	1.626 (1.140–2.319)
GBS	7 (0–12)		13 (6–18)	1.967 (1.487–2.602)	2.116 (1.537–2.915)
AIMS65 score	1 (0–2)		2 (0–5)	2.619 (1.494–4.592)	3.889 (1.880–8.043)
Rockall Score (Pre-endoscopy)	3 (0–5)		4 (2–7)	2.040 (1.283–3.245)	2.931 (1.609–5.340)
Shock index	0.74 (0.42–1.13)		0.97 (0.26–1.88)	22.516 (3.194–158.725)	21.796 (2.944–161.351)

*OR* Odds ratio, *CI* Confidence interval, *MEWS* Modified early warning score, *MELD* Model for end-stage liver disease, *GBS* Glasgow-blatchford score

^a^Controlling for centered age, sex, and recent hemorrhage after endoscopy

**Table 5:** The association of factors for mortality in patients with variceal bleeding

Factor	Survival (*N* = 107)	Mortality (*N* = 11)	Mortality	
			Crude OR (95% CI)	Adjust OR^a^ (95% CI)
D-dimer to albumin ratio > 450, n (%)	35 (32.7%)	10 (90.9%)	20.571 (2.532–167.140)	26.261 (3.054–225.827)
MEWS	2 (1–10)	4 (1–8)	1.387 (1.049–1.833)	1.370 (1.033–1.816)
MELD	14 (7–30)	21 (10–37)	1.190 (1.076–1.317)	1.185 (1.064–1.320)
Child–Pugh score	7 (5–14)	11 (6–12)	1.774 (1.291–2.436)	1.799 (1.269–2.550)
GBS	12 (0–18)	14 (10–16)	1.190 (1.076–1.317)	1.322 (1.028–1.701)
AIMS65 score	1 (0–4)	2 (0–5)	2.615 (1.467–4.665)	2.745 (1.451–5.192)
Rockall Score (Pre-endoscopy)	4 (0–7)	5 (4–6)	2.982 (1.443–6.159)	3.548 (1.482–8.493)
Shock index	0.88 (0.35–1.88)	1.05 (0.26–1.80)	4.101 (0.737–22.833)	5.150 (0.745–35.617)

*OR* Odds ratio, *CI* Confidence interval, *MEWS* Modified early warning score, *MELD* Model for end-stage liver disease, *GBS* Gasgow-blatchford score

^a^Controlling for centered age, sex, and recent hemorrhage after endoscopy

### Association between the DAR and long-term outcome

**Fig. 3:**
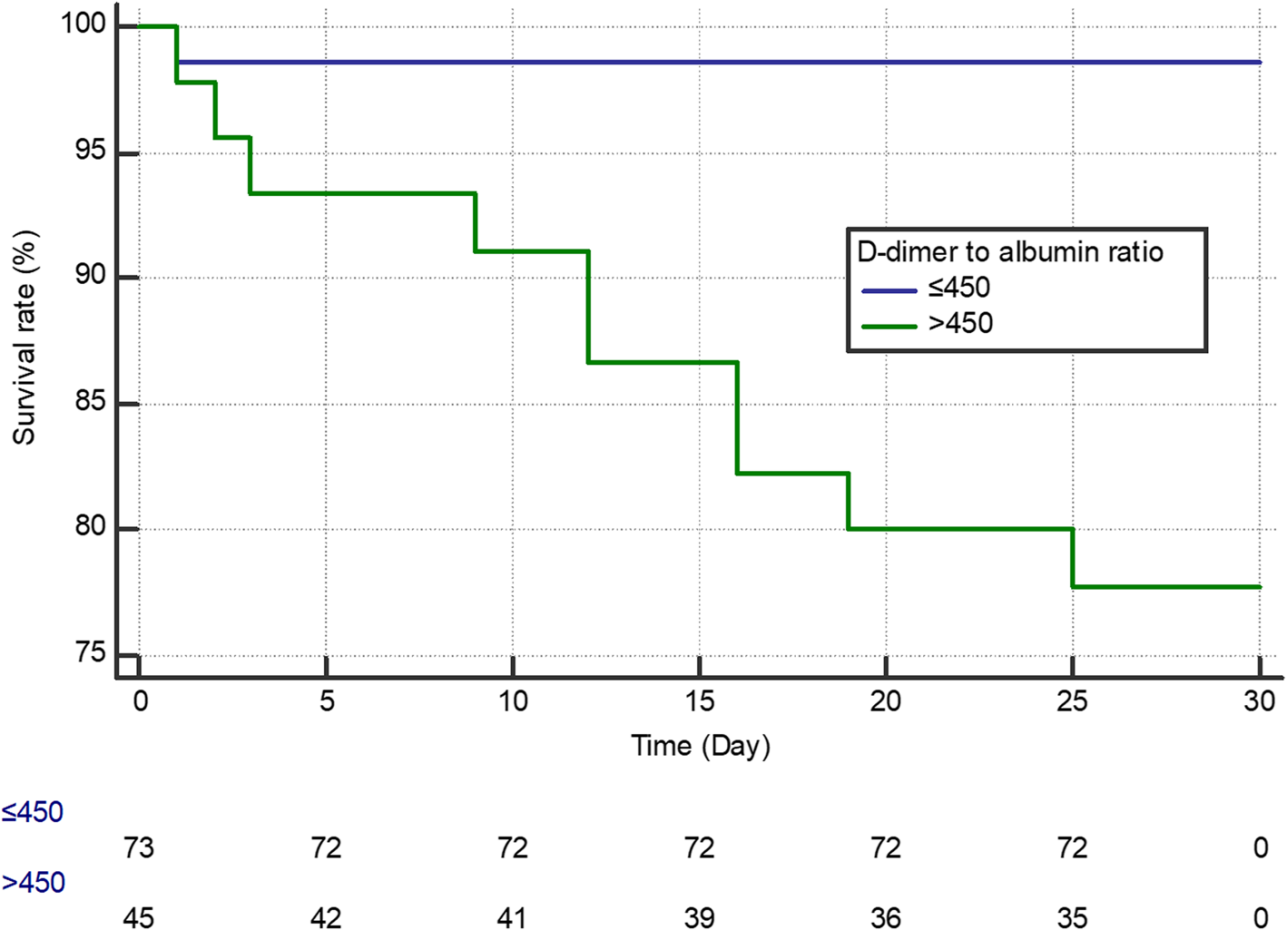
Kaplan–Meier analyses of the time to death

During the observation period, 11 patients died. An observation period of 30 days was established for survival analysis for the DAR cut-off value. All 118 patients were enrolled for survival analysis over 30 days, and patients with a DAR > 450 showed a higher mortality rate than those with a DAR ≤ 450 (Fig. 3).

## Discussion

In this study, we compared and analyzed the various scoring systems for each outcome. In the multivariate logistic regression analysis, the DAR was found to be associated with outcomes such as ICU admission, mortality, long-term hospitalization, and transfusion in patients with AVB, similar to the other scoring systems. To our knowledge, this was the first study to apply the DAR, MELD, and Child–Pugh score and upper gastrointestinal bleeding scoring systems such as the GBS, AIMS65, Pre-endoscopy Rockall score, and MEWS to patients with AVB. The area under the curve for the need for intensive care of the DAR for patients with AVB was 0.695, not reaching higher than fair explanatory power. However, the other scoring systems also did not have an area under the curve of 0.7 or higher, and therefore the DAR showed a relatively high area under the curve compared to the other scoring systems, which may be meaningful. The performance of the DAR as measured in the emergency department cannot be considered satisfactory, with an area under the curve below 0.7 for the need for intensive care and the need for transfusion. However, regarding long-term hospitalization and mortality, the DAR provides fair prediction accuracy, and even if its performance is still suboptimal, it can help the ED physician to understand the severity of the patient’s condition.

The MEWS was applied in this study as a predictive factor for ICU admission. The MEWS can be simply used in the emergency department for critical ill patients, and scores of 5 or higher were associated with increased risk of death and ICU admission [19]. The Simplified Acute Physiology Score and Acute Physiology and Chronic Health Evaluation II are used to predict the need for intensive care [27]. However, in this study, the corresponding scoring system could not be applied. Instead, the MEWS was applied; the MEWS identifies patients at risk of deterioration who require increased levels of care in the ICU [19]. The MEWS is based on physiological values and is simple and practical to use in the ED compared to other scoring systems that require various biomarkers variables [28]. In the case of long hospitalization, the Child–Pugh and MELD scores have proven value for patients with liver cirrhosis [29]. This study was the first to apply the MELD and Child–Pugh scoring systems to examine long hospitalization in patients with AVB. In our study, both scoring systems were confirmed with a good factor of 0.8 or higher. The AIMS65 score was also identified as a good predictive factor for long-term hospitalization. In patients with cirrhosis with upper gastrointestinal bleeding, the AIMS65 score correlated with the length of hospitalization in AVB [30]. In the case of transfusion, the GBS was significantly more valuable than the other scoring systems. As hemoglobin is included in the GBS, if hemoglobin, the recommendation for acute care, is lower than 8, it can be considered that there is need for transfusion [31]. In the case of mortality, the only scoring system that performed well or better based on the area under the curve was the Child–Pugh score. This supports previous findings that the MELD and Child–Pugh scores are survival predictors [20, 32]. In previous studies, the GBS and both Rockall scores were found to be poor at predicting clinical outcomes such as mortality [33]. The area under the curve of the DAR was 0.794, higher than that of the MELD score, and it may be possible to use the DAR to predict mortality, but the number of deaths examined in this study was small, and further studies are needed.

Scoring that can predict poor outcomes in variceal bleeding, which is a disease-specific condition associated with upper gastrointestinal bleeding and cirrhosis, is focused on the prognosis of liver disease, as predicted by the MELD or Child–Pugh scores [2]. Existing upper gastrointestinal bleeding (UGIB) scores require various laboratory data and physical examination findings, such as history, ascites through ultrasonography, cardiac function evaluation, endoscopic findings, and dynamic blood pressure changes, heart rate, and level of consciousness [3]. In this study, as the scoring system in the ED was comparatively analyzed, the post endoscopic Rockall score was excluded from the outcome assessment. All patients underwent endoscopy after admission, and the post-endoscopy Rockall score could not be calculated in the ED.

Early risk stratification is critical in the management of UGIB and may improve patient outcomes and reduce unnecessary healthcare costs through standardization of care [34]. In this study, the cut-off of the DAR by outcome was different, but outcomes other than transfusion generally showed a value of > 400. In a previous study, it was found that D-dimer levels were significantly correlated with the Child–Pugh and MELD scores. The cut-off for in-hospital mortality in variceal bleeding was at D-dimer levels of 280 ng/mL (reference level: 0–300 ng/mL) in previous study [35]. Moreover, D-dimer itself may be elevated in patients with cirrhotic bleeding but showed not significant predictive ability regarding major bleeding complications; the D-dimer levels serving as a measure of fibrin degradation might have limited value because of slow crosslinking associated with deficient factor XIII in cirrhosis [36]. However, this study showed that considering albumin in addition to D-dimer can be useful for various outcome assessments. The cut-off of albumin, which predicts mortality in patients with non-variceal bleeding, was 3.1 in a previous study [37].

### Limitations

First, the number of cases was insufficient to predict mortality. Second, selection bias may have occurred if active treatment was not desired owning to patient needs (terminal stage, economic reasons). The ICU admission criteria may vary depending on the medical environment of each institution. However, criteria based on the priority model used in this cohort are identified as an unstable condition that can be accepted according to general consensus, such as ventilator care, vasoactive drugs, and mental status; therefore, the findings may be generalizable. Third, the reference ranges for D-dimer and albumin may vary across institutions. In addition, there may have been bias depending on the time of visiting the ED. Fourth, the CIs for the odds ratios of the cut-off values of DAR for the different outcomes were relatively wide in our analysis, due to the small sample size; our findings are, however, statistically significant. Fifth, of the 137 patients in this study, 18 did not undergo D-dimer tests, which may have affected the results. Therefore, it is clear that external validation is necessary in future studies.

## Conclusions

The novel biomarker DAR is a useful factor in evaluating the need for intensive care, long-term hospitalization, blood transfusion, and mortality in patients with AVB in the ED, but its explanatory power is not high. Depending on the assessed outcome, the MEWS, Child–Pugh score, MELD score, Rockall score, AIMS65 score, or GBS should be used instead.

## Supplementary Information

**Additional file 1:** **Supplement 1.** Predicting accuracy of the DAR for outcomes. **Supplement 2.** Prediction accuracy of the analyzed laboratory factors for the assessed outcomes.

## Data Availability

The data used to support the findings of this study are available from the corresponding author upon request.

## Abbreviations

AVB: Acute variceal bleeding
ED: Emergency department
DAR: Dimer to albumin ratio
ROC: Receiver operating characteristic
GBS: Glasgow-Blatchford
INR: International normalized ratio
ICU: Intensive care unit
MELD: Model end-stage liver disease
MEWS: Modified early warning score
UGIB: Upper gastrointestinal bleeding
