# Supplementary material for: Usefulness of the d-dimer to albumin ratio for risk assessment in patients with acute variceal bleeding at the emergency department: retrospective observational study
Source: BMC Emerg Med. 2022 Jul 25;22:135. doi: 10.1186/s12873-022-00696-4 (PMC9311345; doi:10.1186/s12873-022-00696-4)
Supplement: Supplementary file 1 — Additional file 1: Supplement 1. Predicting accuracy of the DAR for outcomes. Supplement 2. Prediction accuracy of the analyzed laboratory factors for the assessed outcomes. [file 12873_2022_696_MOESM1_ESM.docx]

**Supplementary Materials**

**Supplement 1**. **Predicting accuracy of the DAR for outcomes**

| Outcomes | AUC  (95% CI) | p-value | Sensitivity  (95% CI) | Specificity  (95% CI) | PPV  (95% CI) | NPV  (95% CI) |
| --- | --- | --- | --- | --- | --- | --- |
| Need for intensive care | 0.695  (0.604–0.777) | <0.001 | 55.22  (42.6–67.4) | 78.43  (64.7–88.7) | 77.1  (65.6–58.6) | 57.1  (49.6–64.3) |
| Need for long-term hospitalization | 0.771  (0.685–0.843) | <0.001 | 84.62  (54.6–98.1) | 65.71  (55.8–74.7) | 23.4  (17.7–30.3) | 97.2  (90.5–99.2) |
| Need for transfusion | 0.679  (0.587–0.762) | <0.001 | 81.05  (71.7–88.4) | 52.17  (30.6–73.2) | 87.5  (81.9–91.6) | 40.0  (27.4–54.1) |
| Predicting mortality | 0.794  (0.709–0.863) | <0.001 | 90.91  (58.7–99.8) | 68.22  (58.5–76.9) | 22.7  (17.4–29.1) | 98.6  (91.8–99.8) |

AUC: area under the curve; CI: confidence interval; PPV: positive predictive value; NPV: negative predictive value

**Supplement 2. Prediction accuracy of the analyzed laboratory factors for the assessed outcomes**

| Factor | Need for intensive care | Need for long term hospitalization | | Need for transfusion | Mortality |
| --- | --- | --- | --- | --- | --- |
|  | AUC (95% CI) | AUC (95% CI) | | AUC (95% CI) | AUC (95% CI) |
| D-dimer, ng/mL | 0.681 (0.589-0.764) | | 0.726 (0.636-0.804) | 0.633 (0.540-0.720) | 0.733 (0.644-0.810) |
| Albumin, g/dL | 0.654 (0.561-0.739) | | 0.786 (0.701-0.856) | 0.833 (0.754-0.896) | 0.788 (0.703-0.858) |
| Hemoglobin, g/dL | 0.698 (0.607-0.779) | | 0.705 (0.614-0.785) | 0.958 (0.905-0.987) | 0.693 (0.601-0.774) |
| BUN, mg/dL | 0.534 (0.440-0.626) | | 0.827 (0.433-0.619) | 0.670 (0.578-0.754) | 0.728 (0.638-0.806) |
| Creatinine, mg/dL | 0.689 (0.597-0.771) | | 0.612 (0.518-0.701) | 0.734 (0.644-0.811) | 0.790 (0.706-0.860) |
| Sodium, mmol/L | 0.577 (0.483-0.668) | | 0.698 (0.607-0.779) | 0.563 (0.469-0.654) | 0.723 (0.634-0.802) |
| INR | 0.631 (0.537-0.718) | | 0.859 (0.782-0.916) | 0.730 (0.641-0.808) | 0.755 (0.667-0.829) |

BUN: blood urea nitrogen; INR: international normalized ratio; AUC: area under the curve; CI: confidence interval
